# Robust multi-tissue gene panel for cancer detection

**DOI:** 10.1186/1471-2407-10-319

**Published:** 2010-06-22

**Authors:** Joseph Irgon, C Chris Huang, Yi Zhang, Dmitri Talantov, Gyan Bhanot, Sándor Szalma

**Affiliations:** 1 Centocor R&D, Inc., 145 King of Prussia Rd, Radnor, PA, 19087, USA; 2 Centocor R&D, Inc., 3210 Merryfield Row, San Diego, CA, 92121, USA; 3 Veridex, LLC, 3210 Merryfield Row, San Diego, CA, 92121, USA; 4 Johnson & Johnson Pharmaceutical Research & Development, L.L.C., 3210 Merryfield Row, San Diego, CA, 92121, USA; 5 Institute for Advanced Study, Einstein Drive, Princeton, NJ, 08540, USA; 6 The Cancer Institute of New Jersey, New Brunswick, NJ, 08901, USA; 7 BioMaps Institute, Rutgers University, New Brunswick, NJ, 08901, USA; 8 Department of Molecular Biology & Biochemistry and Department of Physics, Rutgers University, New Brunswick, NJ, 08901, USA

## Abstract

**Background:**

We have identified a set of genes whose relative mRNA expression levels in various solid tumors can be used to robustly distinguish cancer from matching normal tissue. Our current feature set consists of 113 gene probes for 104 unique genes, originally identified as differentially expressed in solid primary tumors in microarray data on Affymetrix HG-U133A platform in five tissue types: breast, colon, lung, prostate and ovary. For each dataset, we first identified a set of genes significantly differentially expressed in tumor vs. normal tissue at p-value = 0.05 using an experimentally derived error model. Our common cancer gene panel is the intersection of these sets of significantly dysregulated genes and can distinguish tumors from normal tissue on all these five tissue types.

**Methods:**

Frozen tumor specimens were obtained from two commercial vendors Clinomics (Pittsfield, MA) and Asterand (Detroit, MI). Biotinylated targets were prepared using published methods (Affymetrix, CA) and hybridized to Affymetrix U133A GeneChips (Affymetrix, CA). Expression values for each gene were calculated using Affymetrix GeneChip analysis software MAS 5.0. We then used a software package called Genes@Work for differential expression discovery, and SVM light linear kernel for building classification models.

**Results:**

We validate the predictability of this gene list on several publicly available data sets generated on the same platform. Of note, when analysing the lung cancer data set of Spira et al, using an SVM linear kernel classifier, our gene panel had 94.7% leave-one-out accuracy compared to 87.8% using the gene panel in the original paper. In addition, we performed high-throughput validation on the Dana Farber Cancer Institute GCOD database and several GEO datasets.

**Conclusions:**

Our result showed the potential for this panel as a robust classification tool for multiple tumor types on the Affymetrix platform, as well as other whole genome arrays. Apart from possible use in diagnosis of early tumorigenesis, some other potential uses of our methodology and gene panel would be in assisting pathologists in diagnosis of pre-cancerous lesions, determining tumor boundaries, assessing levels of contamination in cell populations in vitro and identifying transformations in cell cultures after multiple passages. Moreover, based on the robustness of this gene panel in identifying normal vs. tumor, mislabelled or misinterpreted samples can be pinpointed with high confidence.

## Background

Rapid and accurate classification of cancerous tissue samples is an unmet scientific and clinical need. Standard clinical practice in identifying cancer relies on pathological examination of biopsy specimens, radiological images and histology. However, these diagnoses can be incorrect because of atypical morphologies, or poorly extracted biopsies. In cases where the pathologist makes an error in determining whether a surgically resected tumor has sufficient normal cells in its margins, an error could have significant consequences to the patient. A corroboratory analysis may also benefit laboratory experiments on cell lines or tissue samples which might be labelled as cancerous, but might in fact be significantly or wholly contaminated by surrounding or externally derived non-cancerous tissue.

Several previous studies have attempted to find a common gene signature in multiple neoplasms. One such group at the NIH has also established a gene panel capable of distinguishing benign from malignant tumor in four different tissue types [1]. In terms of diagnosing cancer from normal specifically, two groups from Johns Hopkins [2,3] have used different methods to analyse the data being collected by ONCOMINE http://www.oncomine.com and have attempted to establish a multi-tissue cancer signature and have claimed and demonstrated success in classifying cancer from normal tissue. The main difference between these two approaches is the algorithm used for feature extraction. Xu et al [4] used a method called top-scoring pair of groups (TSPG) to select informative genes which relies on a random sub sampling of genes. Rhodes et al [2] used a more classical approach to determine the most significantly differentially expressed genes that treats each gene as an independent feature in the dataset. We also use the t-statistic to determine differential expression, which is similar to Rhodes et al [2], but do not assume an underlying normal distribution. Instead, we used an experimentally derived error model for Affymetrix chips incorporated in the Genes@Work software suite from IBM Research which is freely available at: http://www.research.ibm.com/FunGen/FGDownloads.htm. The experimental model used in Genes@Work determines p-values based on a multi-tissue model derived from replicate measurements on Affymetrix chips to assess stochastic and systematic (handling) errors in microarray data analysis.

Our training set consists of a proprietary sample set for normal and cancerous tissue from breast, colon, lung, prostate and ovary. A detailed description of this data is available in the methods section. Using this high quality multi-tissue data set, we applied an integrated informatics strategy which combined targeted bioinformatics and analytical approaches to identify and validate a panel of genes to distinguish normal from cancer tissue. We also demonstrated that an accurate diagnosis of cancer tissue is possible using modern gene expression arrays.

## Methods

### Training set sample and microarray data generation

Frozen tumor specimens were obtained from two commercial vendors Clinomics (Pittsfield, MA) and Asterand (Detroit, MI). The data was obtained from five tissue types (Prostate: 7 Benign, 2 Normal, 10 Cancer), (Lung: 37 Normal, 29 Cancer), (Ovarian: 24 Normal, 22 Cancer), (Colon: 4 Benign, 4 Normal, 33 Cancer), (Breast: 6 Benign, 10 Normal, 31 Cancer) [Table 1]. Total RNA was extracted from 20 to 40 30 μm cryostat sections of tumor tissues (median 90 mg; range, 40-120 mg) with RNAzol B (Campro Scientific, Veenendaal, Netherlands). The median RNA yield was 82 μg (range, 19-240 μg). RNA quality was checked by use of the Agilent BioAnalyzer, and samples were profiled only if they had clear distinct 18S and 28S peaks with no minor peaks present, the area under the 18S and 28S peaks was more than 15% of the total RNA area, and if the 28S/18S ratio was between 1.2 and 2.0. Biotinylated targets were prepared using published methods (Affymetrix, CA) [4] and hybridized to Affymetrix U133A GeneChips (Affymetrix, CA). Expression values for each gene were calculated using Affymetrix GeneChip analysis software MAS 5.0. Chips were rejected if average intensity was < 40 or if the background signal > 100. For normalization, all probe sets were scaled to a target intensity of 600 and scale mask files were not selected.

**Table 1:** Sample distribution for each tissue, and number of differentially expressed genes as calculated by Genes@Work at p < 0.05 [5]

Tissue type	Sample distribution	# of Differentially Expressed Gene probes
Prostate	7 Benign, 2 Normal, 10 Cancer	2035

Lung	37 Normal, 29 Cancer	1961

Ovarian	24 Normal, 22 Cancer	2717

Colon	4 Benign, 4 Normal, 33 Cancer	4159

Breast	6 Benign, 10 Normal, 31 Cancer	2704

Intersection	77 Normal, 125 Cancer	113

### Mapping Affymetrix probes to Agilent probes

Affymetrix probes were first mapped to UniGene Ids using their publically available annotation table and then subsequently mapped to Agilent probes using their respective annotation table. Since many of the genes in our panel are represented by multiple probes, the average expression of each probe was measured across all samples and the probe with the highest overall signal for each gene in our panel was selected as the equivalent diagnostic feature.

### Description of the Genes@Work Software

We used a software package called Genes@Work [5] created by IBM Research, to determine differentially expressed genes in each tissue type. This software uses an experimentally validated non-linear error model for gene expression measurement error derived from replicate measurements which underemphasizes the significance of variations in genes that have lower expression and overemphasizes the significance of variations in genes that have high expression.

### SVM Classifiers

All classifiers were built using SVM light http://svmlight.joachims.org/ using a linear kernel option and complete leave-one-out estimations were calculated for each experiment. M-fold cross validation was also performed at m = 5 and 300 samplings with replacement (Table 2). Normalized data for each of the tissue types were separated into normal and cancer classes and designated as positive and negative classes respectively. Classifiers were built for each tissue type separately as well as globally and saved as SVM model files with support vector information for future classification.

**Table 2:** Leave-one-out and m-fold CV accuracies for normal-tumor classification in our training data using our panel and a linear SVM classifier

Tissue Type	Training LOO Accuracy	M-fold CV Accuracy
Breast	94.73%	92.7%

Colon	96.41%	95.5%

Prostate	100.00%	99.9%

Lung	95.80%	97.3%

Ovary	96.20%	98.2%

Global	91.4%	90.9%

## Results

### Generating a common gene panel for cancer classification

**Figure 1:**
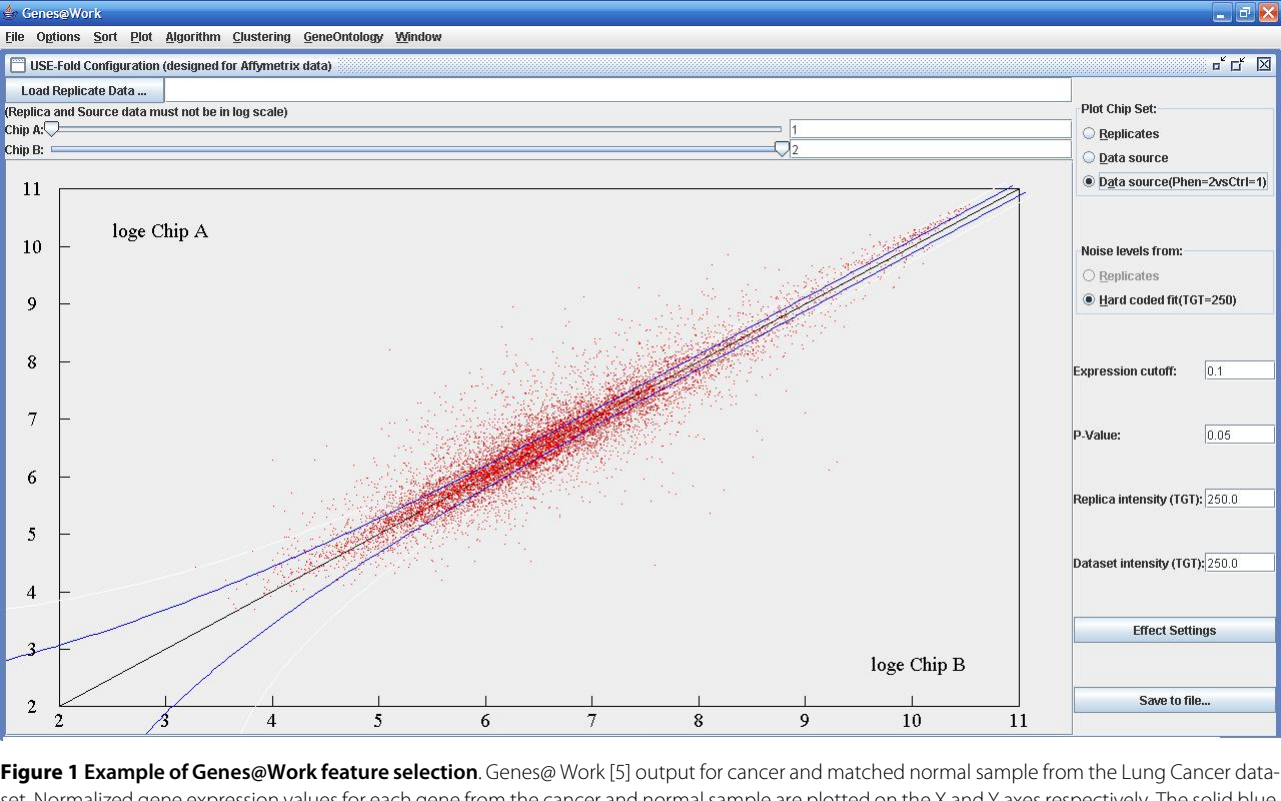
**Example of Genes@Work feature selection**. Genes@ Work [5] output for cancer and matched normal sample from the Lung Cancer dataset. Normalized gene expression values for each gene from the cancer and normal sample are plotted on the X and Y axes respectively. The solid blue lines defines statistical significance boundaries at p = 0.05 from the experimental error model. Red points lying outside above and below the solid lines are gene expression values which are significantly different at p < 0.05 between cancer and normal samples.

In order to explore the possibility of a common gene panel that can reliably detect cancer originated from multiple tissue types, we created a compendia of 5 microarray datasets from prostate, lung, ovarian, colon, and breast, respectively, each with cancer and normal samples from multiple subjects (Table 1). Primary tumor samples and normal samples were collected and processed on Affymetrix HG-U133A GeneChip and subsequently RMA normalized and uploaded to an internal database. The raw intensity data was also log2 transformed, normalized and then input to Genes@Work. This software uses an error model based on replicate Affymetrix chip measurements to determine the true error bounds and p-values and was therefore was an ideal choice for this type of analysis. Figure 1 shows an example of the program output. Points outside of the lines correspond to genes that are differentially expressed with a p-value < 0.05. We generated one set of significantly dysregulated genes for each tissue type by comparing normal samples to tumor samples in that tissue type. Table 1 shows the distribution of samples and the corresponding differentially expressed genes.

**Figure 2:**
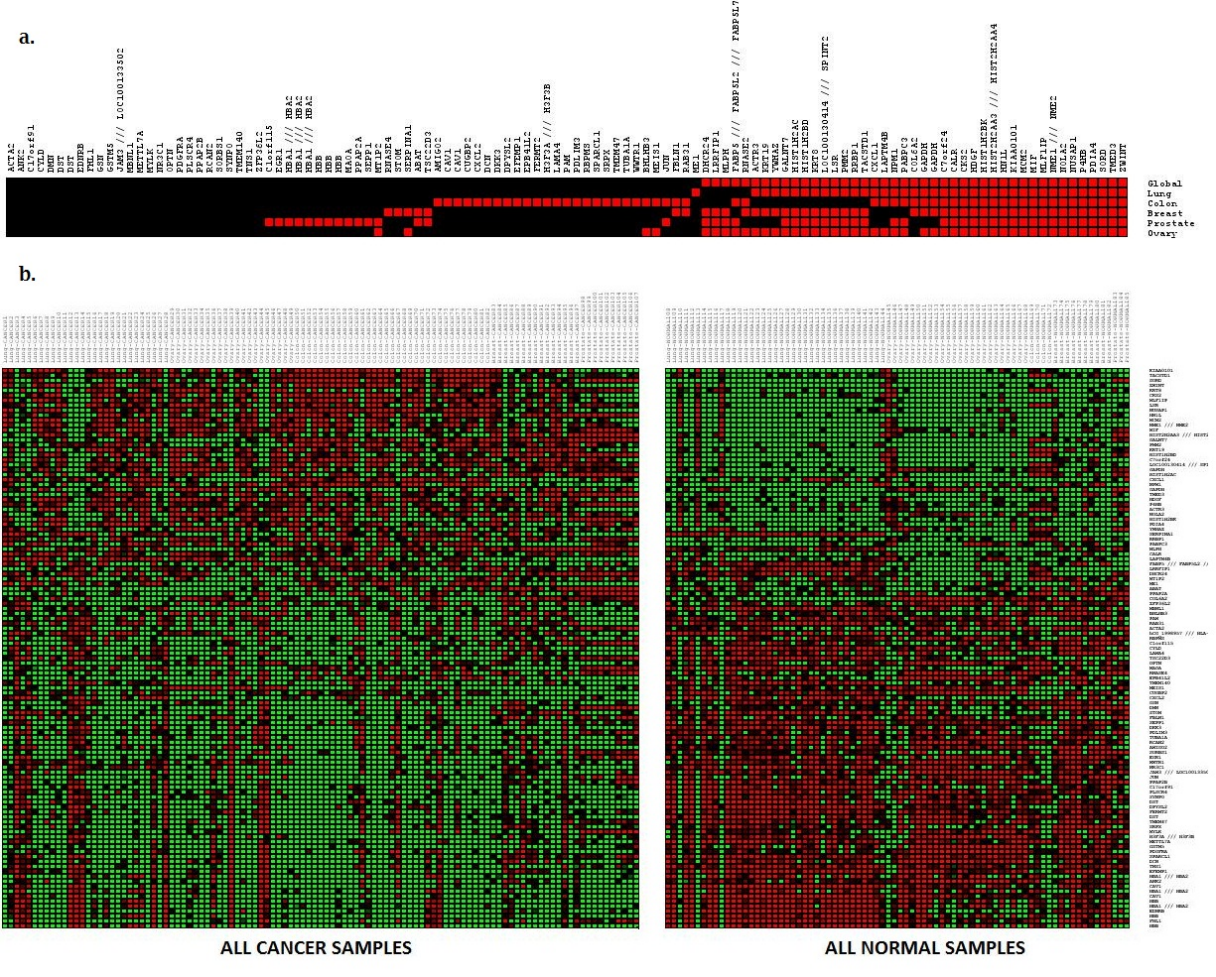
**Expression and signature trends of the training data**. **2a **- Signature trends of each tissue type. Red designates genes that are on average over-expressed when compared to their tissue's average normal expression. Black designates under expression versus normal. **2b **- Combined training data for Lung, Breast, Ovarian, Prostate and Colon tissue samples grouped by phenotype and ranked by the ratio of differential gene expression. Tumors are on the left and normals are on the right.

Next, we identified the intersection of all genes differentially expressed in all five tissue types, resulting in our common cancer gene panel. Using leave-one-out (LOO) cross validation as well as m-fold cross validation, we verified that our common "tumor discriminating" gene panel was robust and could separate cancerous tissue from normal tissue with accuracies exceeding 90% when the tissue of origin was known (see Table 2). Figure 2a shows the relative expressions of each gene in our common feature set in cancer and corresponding normal samples for each tissue type. This figure also shows the expression trends of each of the genes in our panel. A binary table representing this information including all probe ids used is available in Table 3. Figure 2b depicts entire data set grouped by phenotype (cancer versus normal) and demonstrates the striking distinction in gene expression of the panel genes between cancer and normal tissue. To validate that our common caner signature can correctly and robustly classify tumors, we applied our panel to data from several published studies on tumors originating from different tissue types. The results are summarized in Table 4, and described in detail below.

**Table 3:** Gene Names, Affymetrix Probe Ids and their corresponding signatures in each tissue type

Genes	Affy ID	Global	Lung	Colon	Breast	Prostate	Ovary
ABAT	209459_s_at	*-1*	*-1*	*-1*	**1**	**1**	*-1*
ACTA2	200974_at	*-1*	*-1*	*-1*	*-1*	*-1*	*-1*
AMIGO2	222108_at	*-1*	*-1*	**1**	*-1*	*-1*	*-1*
ANK2	202920_at	*-1*	*-1*	*-1*	*-1*	*-1*	*-1*
BHLHB3	221530_s_at	*-1*	*-1*	**1**	*-1*	*-1*	**1**
C17orf91	214696_at	*-1*	*-1*	*-1*	*-1*	*-1*	*-1*
C1orf115	218546_at	*-1*	*-1*	*-1*	*-1*	**1**	*-1*
CAV1	203065_s_at	*-1*	*-1*	**1**	*-1*	*-1*	*-1*
CAV1	203065_s_at	*-1*	*-1*	**1**	*-1*	*-1*	*-1*
CUGBP2	202158_s_at	*-1*	*-1*	**1**	*-1*	*-1*	*-1*
CXCL2	209774_x_at	*-1*	*-1*	**1**	*-1*	*-1*	*-1*
CYLD	39582_at	*-1*	*-1*	*-1*	*-1*	*-1*	*-1*
DCN	201893_x_at	*-1*	*-1*	**1**	*-1*	*-1*	*-1*
DKK3	214247_s_at	*-1*	*-1*	**1**	*-1*	*-1*	*-1*
DMN	212730_at	*-1*	*-1*	*-1*	*-1*	*-1*	*-1*
DPYSL2	200762_at	*-1*	*-1*	**1**	*-1*	*-1*	*-1*
DST	212254_s_at	*-1*	*-1*	*-1*	*-1*	*-1*	*-1*
DST	212254_s_at	*-1*	*-1*	*-1*	*-1*	*-1*	*-1*
EDNRB	204273_at	*-1*	*-1*	*-1*	*-1*	*-1*	*-1*
EFEMP1	201842_s_at	*-1*	*-1*	**1**	*-1*	*-1*	*-1*
EGR1	201694_s_at	*-1*	*-1*	*-1*	*-1*	**1**	*-1*
EPB41L2	201719_s_at	*-1*	*-1*	**1**	*-1*	*-1*	*-1*
FBLN1	202995_s_at	*-1*	*-1*	**1**	**1**	*-1*	*-1*
FERMT2	209210_s_at	*-1*	*-1*	**1**	*-1*	*-1*	*-1*
FHL1	201540_at	*-1*	*-1*	*-1*	*-1*	*-1*	*-1*
GSN	200696_s_at	*-1*	*-1*	*-1*	*-1*	*-1*	*-1*
GSTM5	205752_s_at	*-1*	*-1*	*-1*	*-1*	*-1*	*-1*
H3F3A///H3F3B	211998_at	*-1*	*-1*	**1**	*-1*	*-1*	*-1*
HBA1///HBA2	209458_x_at	*-1*	*-1*	*-1*	*-1*	**1**	*-1*
HBA1///HBA2	209458_x_at	*-1*	*-1*	*-1*	*-1*	**1**	*-1*
HBA1///HBA2	209458_x_at	*-1*	*-1*	*-1*	*-1*	**1**	*-1*
HBB	209116_x_at	*-1*	*-1*	*-1*	*-1*	**1**	*-1*
HBB	209116_x_at	*-1*	*-1*	*-1*	*-1*	**1**	*-1*
HBB	209116_x_at	*-1*	*-1*	*-1*	*-1*	**1**	*-1*
JAM3///LOC100133502	212813_at	*-1*	*-1*	*-1*	*-1*	*-1*	*-1*
JUN	201466_s_at	*-1*	*-1*	**1**	*-1*	**1**	*-1*
LAMA4	202202_s_at	*-1*	*-1*	**1**	*-1*	*-1*	*-1*
MAOA	212741_at	*-1*	*-1*	*-1*	*-1*	**1**	*-1*
MBNL1	201153_s_at	*-1*	*-1*	*-1*	*-1*	*-1*	*-1*
ME1	204059_s_at	*-1*	**1**	*-1*	*-1*	*-1*	*-1*
MEIS1	204069_at	*-1*	*-1*	**1**	*-1*	*-1*	**1**
METTL7A	207761_s_at	*-1*	*-1*	*-1*	*-1*	*-1*	*-1*
MT1P2	211456_x_at	*-1*	*-1*	*-1*	*-1*	**1**	**1**
MYLK	202555_s_at	*-1*	*-1*	*-1*	*-1*	*-1*	*-1*
NR3C1	211671_s_at	*-1*	*-1*	*-1*	*-1*	*-1*	*-1*
OPTN	202073_at	*-1*	*-1*	*-1*	*-1*	*-1*	*-1*
PAM	202336_s_at	*-1*	*-1*	**1**	*-1*	*-1*	*-1*
PDGFRA	203131_at	*-1*	*-1*	*-1*	*-1*	*-1*	*-1*
PDLIM3	209621_s_at	*-1*	*-1*	**1**	*-1*	*-1*	*-1*
PLSCR4	218901_at	*-1*	*-1*	*-1*	*-1*	*-1*	*-1*
PPAP2A	210946_at	*-1*	*-1*	*-1*	*-1*	**1**	*-1*
PPAP2B	212230_at	*-1*	*-1*	*-1*	*-1*	*-1*	*-1*
RAB31	217762_s_at	*-1*	*-1*	**1**	**1**	*-1*	*-1*
RBPMS	209487_at	*-1*	*-1*	**1**	*-1*	*-1*	*-1*
RCAN2	203498_at	*-1*	*-1*	*-1*	*-1*	*-1*	*-1*
RNASE4	205158_at	*-1*	*-1*	*-1*	**1**	*-1*	*-1*
SEPP1	201427_s_at	*-1*	*-1*	*-1*	*-1*	**1**	*-1*
SERPINA1	211429_s_at	*-1*	*-1*	*-1*	**1**	*-1*	**1**
SORBS1	218087_s_at	*-1*	*-1*	*-1*	*-1*	*-1*	*-1*
SPARCL1	200795_at	*-1*	*-1*	**1**	*-1*	*-1*	*-1*
SRPX	204955_at	*-1*	*-1*	**1**	*-1*	*-1*	*-1*
STOM	201060_x_at	*-1*	*-1*	*-1*	**1**	*-1*	*-1*
SYNPO	202796_at	*-1*	*-1*	*-1*	*-1*	*-1*	*-1*
TMEM140	218999_at	*-1*	*-1*	*-1*	*-1*	*-1*	*-1*
TMEM47	209656_s_at	*-1*	*-1*	**1**	*-1*	*-1*	*-1*
TNS1	221748_s_at	*-1*	*-1*	*-1*	*-1*	*-1*	*-1*
TSC22D3	208763_s_at	*-1*	*-1*	*-1*	**1**	**1**	*-1*
TUBA1A	209118_s_at	*-1*	*-1*	**1**	*-1*	*-1*	*-1*
WWTR1	202133_at	*-1*	*-1*	**1**	*-1*	*-1*	*-1*
ZFP36L2	201368_at	*-1*	*-1*	*-1*	*-1*	*-1*	*-1*
ACTR3	200996_at	**1**	**1**	*-1*	**1**	*-1*	**1**
C7orf24	215380_s_at	**1**	**1**	**1**	**1**	**1**	**1**
CALR	214315_x_at	**1**	**1**	**1**	**1**	**1**	**1**
CKS2	204170_s_at	**1**	**1**	**1**	**1**	**1**	**1**
COL6A2	209156_s_at	**1**	**1**	**1**	**1**	*-1*	*-1*
CXCL1	204470_at	**1**	**1**	**1**	*-1*	*-1*	**1**
DHCR24	200862_at	**1**	*-1*	*-1*	**1**	**1**	**1**
FABP5///FABP5L2///FABP5L7	202345_s_at	**1**	*-1*	**1**	*-1*	**1**	**1**
GALNT7	218313_s_at	**1**	**1**	*-1*	**1**	**1**	**1**
GAPDH	M33197_5_at	**1**	**1**	**1**	**1**	*-1*	**1**
GAPDH	M33197_5_at	**1**	**1**	**1**	**1**	*-1*	**1**
HDGF	200896_x_at	**1**	**1**	**1**	**1**	**1**	**1**
HIST1H2AC	215071_s_at	**1**	**1**	*-1*	**1**	**1**	**1**
HIST1H2BD	209911_x_at	**1**	**1**	*-1*	**1**	**1**	**1**
HIST1H2BK	209806_at	**1**	**1**	**1**	**1**	**1**	**1**
HIST2H2AA3///HIST2H2AA4	214290_s_at	**1**	**1**	**1**	**1**	**1**	**1**
HN1L	212115_at	**1**	**1**	**1**	**1**	**1**	**1**
KIAA0101	202503_s_at	**1**	**1**	**1**	**1**	**1**	**1**
KRT19	201650_at	**1**	**1**	*-1*	**1**	*-1*	**1**
KRT8	209008_x_at	**1**	**1**	*-1*	**1**	**1**	**1**
LAPTM4B	214039_s_at	**1**	**1**	**1**	*-1*	*-1*	**1**
LOC100130414///SPINT2	210715_s_at	**1**	**1**	*-1*	**1**	**1**	**1**
LRRFIP1	201861_s_at	**1**	*-1*	*-1*	**1**	**1**	**1**
LSR	208190_s_at	**1**	**1**	*-1*	**1**	**1**	**1**
MCM2	202107_s_at	**1**	**1**	**1**	**1**	**1**	**1**
MIF	217871_s_at	**1**	**1**	**1**	**1**	**1**	**1**
MLF1IP	218883_s_at	**1**	**1**	**1**	**1**	**1**	**1**
MLPH	218211_s_at	**1**	*-1*	*-1*	**1**	**1**	**1**
NME1///NME2	201577_at	**1**	**1**	**1**	**1**	**1**	**1**
NOLA2	209104_s_at	**1**	**1**	**1**	**1**	**1**	**1**
NPM1	221923_s_at	**1**	**1**	**1**	*-1*	**1**	**1**
NUSAP1	218039_at	**1**	**1**	**1**	**1**	**1**	**1**
P4HB	200654_at	**1**	**1**	**1**	**1**	**1**	**1**
PABPC3	208113_x_at	**1**	**1**	**1**	*-1*	**1**	**1**
PDIA4	211048_s_at	**1**	**1**	**1**	**1**	**1**	**1**
PMM2	203201_at	**1**	**1**	*-1*	**1**	**1**	**1**
RNASE2	215193_x_at	**1**	*-1*	**1**	**1**	*-1*	**1**
RRBP1	201204_s_at	**1**	**1**	*-1*	**1**	**1**	**1**
SORD	201563_at	**1**	**1**	**1**	**1**	**1**	**1**
TACSTD1	201839_s_at	**1**	**1**	*-1*	**1**	**1**	**1**
TMED3	208837_at	**1**	**1**	**1**	**1**	**1**	**1**
YWHAZ	200641_s_at	**1**	**1**	*-1*	**1**	*-1*	**1**
ZWINT	204026_s_at	**1**	**1**	**1**	**1**	**1**	**1**

1 (Blue) represents upregulated with respect to normal and -1 (Red) downregulated.

**Table 4:** Leave-one-out and validation accuracies for normal-tumor classification in various tissue types using our panel and a linear SVM classifier

Data Set	Accuracy
Multi-tissue SVM on Wang Data[5]	100%

Breast Cancer Metastasis Training [5]	88.80%

Multi-tissue SVM on Ovarian Cancer GCOD (2 studies) [7,8]	100%

Multi-tissue SVM on Colon Cancer GCOD (2 studies) [9,10]	100%

Other tissue validation sets (Bladder, Melanoma, ccRCC) [11,12]	95.4%

### Validation set #1: Lung Cancer

**Figure 3:**
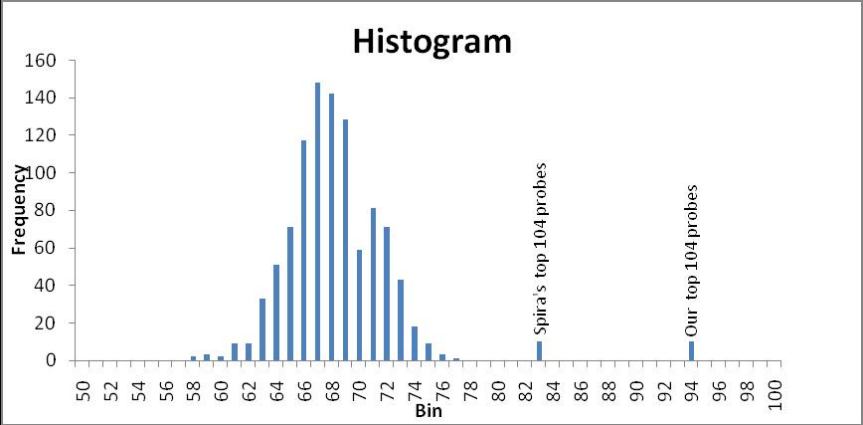
**Distribution of LOO accuracies**. Distribution of LOO accuracies using randomly selected gene lists to classify Lung cancer from the dataset of Spira et al. [6], overlayed with accuracies using the dataset from [6] compared to our gene panel.

Spira et al identified an 80 gene panel which, together with cytopathology, was able to distinguish smokers with and without stage 1 lung cancer using bronchial epithelial brushing samples [6]. Using a weighted voting algorithm, they were able to achieve 80% accuracy which improved to 95% when these predictions were combined with cytopathology. In comparison, our gene panel applied to their data and trained on an SVM classifier (without using cytopathology) was able to achieve 94.75% leave-one-out accuracy using the microarray data alone. To make a more direct comparison, we also built SVM classifiers based on Spira's 80 gene probes, as well as the top 104 probes using the same feature extraction methodology that they used. Figure 3 shows the distributions of accuracies for classifiers based on a random choice of genes compared with our panel as well as Spira's original panel described in the publication.

Although the data of Spira et al had samples from bronchial airway epithelial cells and our lung data was for primary lung cancer, the tumor samples in the datasets had similar signatures with respect to the genes in our panel. This was verified by checking that both SVM classifiers using our gene panel, built on the Spira data and tested on primary lung cancer data or vice versa, had classification accuracy > 90%.

### Validation set #2: Breast Cancer

Wang et al published a study with a 286 sample data set of breast cancer samples, 180 from patients who eventually developed distant metastasis within 5 years and 106 from patients who did not [7]. Using both a breast specific and the multi-tissue tissue SVM classifier, we classified all 286 of these samples as cancerous. Further, although we did not expect a positive result, as an exploratory measure we built a classifier that attempted to distinguish between metastatic tumors and non-metastatic lesions. Our classifier achieved 88.8% LOO accuracy, suggesting that although it was developed to distinguish tumors from normal, some of these genes seem to also carry information about the metastatic properties of these tumors.

### Validation set #3: Ovarian Cancer

Data from the Dana Farber Cancer Institute GCOD dataset repository[8] was selected for high-throughput validation of our cancer signatures. We selected data from ovarian and colon tissue which had data points for all 113 of our probes. The array normalized data was then tested using SVM classifiers trained on our data set. Summaries of the results of this analysis are included in Table 4 and show that our panel is highly accurate.

Study 1: [9]: 103 samples ranging from mucinous, clear cell, serious and endometrioid ovarian carcinomas.

Study 2: [10] 27 samples, 23 cancer, 4 normals.

Using both an ovarian specific classifier & a multi-tissue classifier, we obtained 100% accuracy.

### Validation set #4 Colon cancer

Study 1: Groene et al [11] 36 samples ranging conventional mucinous, serated mucinous, serated non-mucinous.

Study 2: Laiho et al [12]: 37 samples which were all colorectal adenocarcinomas.

Using a colon specific classifier & a multi-tissue classifier we were able to correctly label all cancer samples.

To further test the significance of our gene panel, we used an internal data warehouse called tranSMART (private communication, J. Smart, Centocor R&D, Inc) that takes a gene signature as input and searches all deposited internal and public clinical and non-clinical microarray analyses for similar signatures. When inputting our multi-tissue average signature, the top significant results all refer to normal vs cancer comparisons. Our global signature hit 47% of the cancer vs. normal comparisons which was significant relative to the total distribution that only comprises 5% of the database. We then tested the top publicly available hits to see how accurately we could predict the two classes. Specifically, we tested two datasets containing tissue types outside the scope of our training set.

### Validation set #5: Bladder cancer and Melanoma

The first dataset analysed was a bladder cancer dataset from Dyrskjot et al [13] containing 60 samples, 9 of which were normal and the rest were superficial transitional cell carcinoma with and without surrounding carcinoma in situ. The second [14] had 7 normal skin samples and 45 melanoma samples. An interesting visualization of this dataset is available in Additional File 1. Note that our gene panel demonstrates an incomplete transformation signature of the benign nevi in this data set. An analysis combining these two data sets using our SVM classifier yielded 95.2% LOO accuracy for distinguishing tumor from normal (Table 4).

### Validation set #6: Clear Cell Renal Cell Carcinoma (ccRCC)

To establish how well our panel performed for ccRCC/normal-kidney discrimination we used a previously published dataset which was also used by two other groups [2,3] in testing their gene lists to distinguish ccRCC from normal kidney. We downloaded the data the GSE781 dataset from GEO http://www.ncbi.nlm.nih.gov/gds/?term=GSE781 and applied our SVM classifier on this data to distinguish ccRCC from normal kidney. The results are shown in Table 5. At first pass, we noticed that our gene panel performed poorly on this dataset. TMEV [15] visualization of this dataset using our gene panel revealed that several of the samples with sample headings listed as cancerous very closely resembled normal samples (Additional File 2). Furthermore, a careful analysis of the tumor and normal labels in the original data showed that these three samples were indeed normal. Apparently, these three samples were misinterpreted as tumors in the analysis of Rhodes et al [2] and Xu et al [3](we describe this issue further in the discussion section). After properly labelling these samples as normal, our SVM had 100% testing accuracy in distinguishing ccRCC from normal kidney.

**Table 5:** Comparison with the Rhodes and Xu signatures on the same independent data

		Rhodes Signature		Xu Signature		Our Signature	
	GSE#	Accuracy(%)	P-value	Accuracy(%)	P-value	Accuracy(%)	P-value

Gordon_Lung	GSE2549	91.8	3.48E-07	95.9	1.75E-07	97.8	3.50E-09

Hoffman_Myometrium	GSE593	80	2.06E-01	80	8.33E-02	90	8.00E-04

Lenburg_Kidney	GSE781	76.5	1.01E-01	76.5	1.07E-01	76.5|100*	1.00E-10

Talantov_Skin	GSE3189	94.2	8.97E-07	98.1	3.44E-07	94.2	5.11E-04

Wachi_Lung	GSE3268	100	3.97E-03	100	3.97E-03	100	3.97E-03

Yoon_Soft_Tissue	GSE2719	85.2	5.67E-08	96.3	6.76E-11	94.3	9.50E-07

Overall		89.1	9.28E-26	94.3	9.74E-30	96	1.04E-30

*100% Accuracy achieved after labelling is corrected.

To further test our accuracy on ccRCC we also analyzed a microarray dataset from an on-going collaboration at the University of North Carolina Medical School consisting of 52 ccRCC samples and 18 patient matched normal kidney samples from two studies [16-18]. The data was collected on Agilent G4112F whole genome arrays and is posted on GEO (GSE16449). After mapping our Affymetrix probe features to genes, we created a dataset where the level of each gene was represented as the expression level of the probe (for that gene) with the highest average expression across samples (see methods). This reduced the dataset to 100 genes, on which we applied our SVM analysis methodology to distinguish ccRCC from normal kidney. The SVM also had 100% leave-one-out cross validation accuracy on these samples, which suggests that our panel is not only robust across tissue types but also appears to be platform independent. TMEV [15] visualizations of this dataset is also available in Additional File 3.

## Discussion

For the public datasets we used, the pathological stage is given in the papers cited above. For the proprietary datasets, Additional File 4 details all the staging information we have on the tumor samples. We have identified a robust panel of commonly differentially expressed genes from 77 normal tissues and 125 cancer samples and demonstrated that these features capture the neoplastic elements of the cancer samples in sufficient detail across many tumor types to provide an accurate diagnostic for cancer detection. Some of the genes in our panel have been previously indicated in cancer association studies. For example, KIAA0101 (p15PAF) is a PCNA associated factor which has been shown to be upregulated in tumor samples [19]. This gene as well as two others, NME1 and CKS2, is also present in both the Rhodes and Xu signatures [2,3]. However, there are some unexpected discoveries. For example, we found GAPDH, generally believed to be a housekeeping gene, upregulated in tumors vs. normal in all tissue with the exception of prostate tissue where it had the opposite signature. Our results are in agreement with a recent observation [20] that several housekeeping genes, including GAPDH, have a differential signature in tumors versus normal tissue.

When the gene signature is analysed for its functional classifications, it becomes clear that it is enriched in categories important for cell growth. Among the highly enriched categories include actin cytoskeleton organization, DNA packaging, nucleosome and chromatin assembly, and regulation of blood vessel size.

While the Rhodes and Xu gene panels have demonstrated similar success in diagnostic capabilities our gene panel is more robust both in design and performance (Table 5). Our panel outperformed the panels by Rhodes et al [2] and Xu et al [3] in 4 out of 6 tumor types while performed equally well for the remaining two. Taken as a whole, our panel achieved 96% accuracy. It should be noted, however, that three samples interpreted as ccRCC in the Rhodes et al [2] and Xu et al [3] studies were actually normal samples in the original dataset and thus the sample headings were misleading. For instance, one of the samples interpreted as "tumor" in the Rhodes et al [2] and Xu et al [3] studies had the description: "N4 Renal Cell Carcinoma" in GEO. However, the detailed description in the original data read: "Trizol isolation of total RNA from normal tissue adjacent to Renal Cell Carcinoma", which meant that this sample should have been classified as Normal Kidney. In the analysis done by Rhodes et al [3] and Xu et al [4], these errors were not corrected, which may partly explain their poor results on this dataset. The three samples which are misinterpreted in the GEO database as tumor are sample IDs: GSM12283, GSM12300, GSM12444. After properly labelling these samples as normal, our SVM had 100% testing accuracy in distinguishing ccRCC from normal kidney.

The reason for the robustness of the panel is that we use a methodology (Genes@Work) where the error model for expression levels (noise) is derived from actual replicate measurements [5] rather than from assuming that the underlying error is Gaussian, which is implicit in the error models used in other panels. On a practical level, our panel performs remarkably well in unseen testing datasets, many of which were on tissue types which were outside the tissue sets in the training data. Due to the robustness of our gene panel, it should be particularly useful in accurate identification of mislabelled or misinterpreted samples.

## Conclusions

Our feature set has several potential uses. A common problem of cancer sample collection is that surrounding normal tissue can severely contaminate the sample. Since the tissue of origin is usually known in biopsies, our panel can be used as a tool for rapid determination of the presence or absence of cancer cells as an aid to pathologists. It can also be used to determine contamination in in-vitro experiments.

Expression-based diagnosis and risk assessment is rapidly gaining popularity in the clinic [21] with diagnostic panels using measurements of 10 to 35 genes via qRT-PCR experiments. A possible follow up to our study would be to identify and validate a minimal subset of probes which retain sufficient predictive power and which can be measured using RT-PCR from FFPE sections.

## Abbreviations

SVM: Support Vector Machine; GCOD: Gene Chip Oncology Database; GEO: Gene expression Omnibus; qRT-PCR: quantitative real time-polymerase chain reaction; ccRCC: clear cell renal cell carcinoma; LOO: leave-one-out; FFPE: Formalin-Fixed, Paraffin-Embedded

## Competing interests

C. Chris Huang, Yi Zhang, Dmitri Talantov, Sándor Szalma are all employees of pharmaceutical companies of Johnson & Johnson.

## Authors' contributions

JI drafted the manuscript and conducted the majority of the bioinformatics analysis described. YZ and DT supplied the data and carried out initial analysis. SS helped conceive the study and was the main driver of this research initiative. GB served as graduate mentor for JI and thus provided the majority of the scientific input. GB, SS, CH all contributed to editing of the manuscript as well as providing direction and guidance to JI. All authors have read and approved the final manuscript.

## Pre-publication history

The pre-publication history for this paper can be accessed here:

http://www.biomedcentral.com/1471-2407/10/319/prepub

## Supplementary Material

Additional File 1 **Melanoma data set (GSE 3189) visualization**. Melanoma data set (GSE 3189) gene expression sorted by ratio of gene expression ratio of cancer vs. normal. The middle portion contains nevus samples which are considered benign. Interestingly, they appear to have a mixed signature that is an incomplete transformation from normal to cancer. Click here for file

Additional File 2 **The kidney cancer validation sets**. Samples on the left are normal, right are cancer. The first image represents the Lenburg et al. [19] data set. The mislabelled samples in question are the rightmost 3 samples in the normal subgroup. Click here for file

Additional File 3 **ccRCC data from UNC labs**. This image is of the ccRCC data [15,16] from UNC labs done on Agilent chips. Click here for file

Additional File 4 **Distribution of cancer stage in the training data**. The following table shows the distribution of stages of the cancer samples in our training set. Click here for file

## References

[B1] Basil CF Zhao Y Zavaglia K Jin P Panelli MC Voiculescu S Mandruzzato S Lee HM Seliger B Freedman RS Taylor PR Hu N Zanovello P Marincola FM Wang E Common cancer biomarkers Cancer Res 2006 66 2953 2961 10.1158/0008-5472.CAN-05-3433 16540643

[B2] Rhodes DR Yu J Shanker K Deshpande N Varambally R Ghosh D Barrette T Pandey A Chinnaiyan AM Large-scale meta-analysis of cancer microarray data identifies common transcriptional profiles of neoplastic transformation and progression Proc Natl Acad Sci USA 2004 101 9309 9314 10.1073/pnas.0401994101 15184677 PMC438973

[B3] Xu L Geman D Winslow RL Large-scale integration of cancer microarray data identifies a robust common cancer signature BMC Bioinformatics 2007 8 275 10.1186/1471-2105-8-275 17663766 PMC1950528

[B4] Lipshutz RJ Fodor SP Gingeras TR Lockhart DJ High density synthetic oligonucleotide arrays Nat Genet 1999 21 20 24 10.1038/4447 9915496

[B5] Lepre J Rice JJ Tu Y Stolovitzky G Genes@Work: an efficient algorithm for pattern discovery and multivariate feature selection in gene expression data Bioinformatics 2004 20 1033 1044 10.1093/bioinformatics/bth035 14764572

[B6] Spira A Beane JE Shah V Steiling K Liu G Schembri F Gilman S Dumas YM Calner P Sebastiani P Sridhar S Beamis J Lamb C Anderson T Gerry N Keane J Lenburg ME Brody JS Airway epithelial gene expression in the diagnostic evaluation of smokers with suspect lung cancer Nat Med 2007 13 361 366 10.1038/nm1556 17334370

[B7] Wang Y Klijn JG Zhang Y Sieuwerts AM Look MP Yang F Talantov D Timmermans M Meijer-van Gelder ME Yu J Jatkoe T Berns EM Atkins D Foekens JA Gene-expression profiles to predict distant metastasis of lymph-node-negative primary breast cancer Lancet 2005 365 671 679 15721472 10.1016/S0140-6736(05)17947-1

[B8] Dana Farber GeneChip Oncology Database http://compbio.dfci.harvard.edu/tgi/cgi-bin/tucan/tucan.pl

[B9] Hendrix ND Wu R Kuick R Schwartz DR Fearon ER Cho KR Fibroblast growth factor 9 has oncogenic activity and is a downstream target of Wnt signaling in ovarian endometrioid adenocarcinomas Cancer Res 2006 66 1354 1362 10.1158/0008-5472.CAN-05-3694 16452189

[B10] Jochumsen KM Tan Q Holund B Kruse TA Mogensen O Gene expression in epithelial ovarian cancer: a study of intratumor heterogeneity Int J Gynecol Cancer 2007 17 979 985 10.1111/j.1525-1438.2007.00908.x 17367315

[B11] Groene J Mansmann U Meister R Staub E Roepcke S Heinze M Klaman I Brummendorf T Hermann K Loddenkemper C Pilarsky C Mann B Adams HP Buhr HJ Rosenthal A Transcriptional census of 36 microdissected colorectal cancers yields a gene signature to distinguish UICC II and III Int J Cancer 2006 119 1829 1836 10.1002/ijc.22027 16721809

[B12] Laiho P Kokko A Vanharanta S Salovaara R Sammalkorpi H Jarvinen H Mecklin JP Karttunen TJ Tuppurainen K Davalos V Schwartz S Arango D Mäkinen MJ Aaltonen LA Serrated carcinomas form a subclass of colorectal cancer with distinct molecular basis Oncogene 2007 26 312 320 10.1038/sj.onc.1209778 16819509

[B13] Dyrskjot L Kruhoffer M Thykjaer T Marcussen N Jensen JL Moller K Orntoft TF Gene expression in the urinary bladder: a common carcinoma in situ gene expression signature exists disregarding histopathological classification Cancer Res 2004 64 4040 4048 10.1158/0008-5472.CAN-03-3620 15173019

[B14] Talantov D Mazumder A Yu JX Briggs T Jiang Y Backus J Atkins D Wang Y Novel genes associated with malignant melanoma but not benign melanocytic lesions Clin Cancer Res 2005 11 7234 7242 10.1158/1078-0432.CCR-05-0683 16243793

[B15] Saeed AI Bhagabati NK Braisted JC Liang W Sharov V Howe EA Li J Thiagarajan M White JA Quackenbush J TM4 microarray software suite Methods Enzymol 2006 411 134 193 10.1016/S0076-6879(06)11009-5 16939790

[B16] Liu H Brannon AR Reddy A Alexe G Seiler M Arreola A Oza J Yao M Juan D Liou L Ganesan S Levine AJ Rathmell WK Bhanot G Identifying direct mRNA targets of microRNA dysregulated in cancer: with application to clear cell Renal Cell Carcinoma BMC Systems Biology 2010 4 51 10.1186/1752-0509-4-51 20420713 PMC2876063

[B17] Brannon AR Reddy A Seiler M Arreola A Moore DT Pruthi RS Wallen EM Nielsen ME Liu H Ljungberg B Zhao H Brooks JD Nathanson KL Ganesan S Bhanot G Rathmell WK Molecular Stratification of Clear Cell Renal Cell Carcinoma by Consensus Clustering Reveals Distinct Subtypes and Survival Patterns Genes and Cancer 2010 1 2 152 63 10.1177/1947601909359929 PMC2943630 20871783

[B18] Lenburg ME Liou LS Gerry NP Frampton GM Cohen HT Christman MF Previously unidentified changes in renal cell carcinoma gene expression identified by parametric analysis of microarray data BMC Cancer 2003 3 31 10.1186/1471-2407-3-31 14641932 PMC317310

[B19] Yu P Huang B Shen M Lau C Chan E Michel J Xiong Y Payan DG Luo Y p15(PAF), a novel PCNA associated factor with increased expression in tumor tissues Oncogene 2001 20 484 489 10.1038/sj.onc.1204113 11313979

[B20] Byun J Logothetis CJ Gorlov IP Housekeeping genes in prostate tumorigenesis Int J Cancer 2009 125 2603 2608 10.1002/ijc.24680 19551858 PMC4531049

[B21] Slodkowska EA Ross JS MammaPrint 70-gene signature: another milestone in personalized medical care for breast cancer patients Expert Rev Mol Diagn 2009 9 417 422 10.1586/erm.09.32 19580427

