# Supplementary figures and images for: Robust multi-tissue gene panel for cancer detection
Source: BMC Cancer. 2010 Jun 22;10:319. doi: 10.1186/1471-2407-10-319 (PMC2906482; doi:10.1186/1471-2407-10-319)

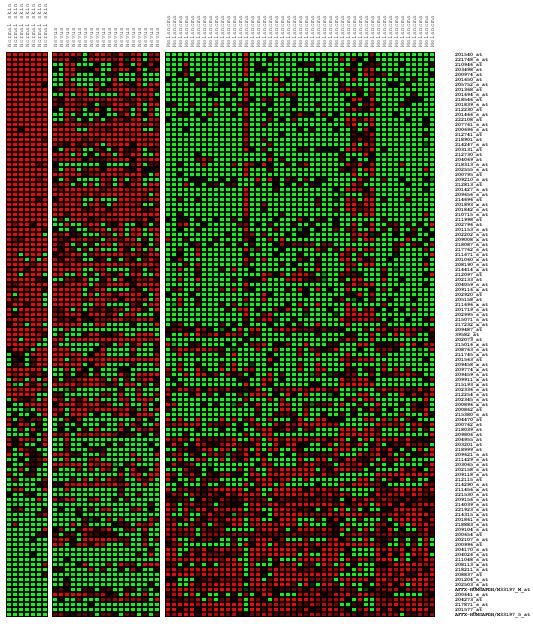

Supplement: Additional File 1 — Melanoma data set (GSE 3189) visualization. Melanoma data set (GSE 3189) gene expression sorted by ratio of gene expression ratio of cancer vs. normal. The middle portion contains nevus samples which are considered benign. Interestingly, they appear to have a mixed signature that is an incomplete transformation from normal to cancer. [file 1471-2407-10-319-S1.JPEG]

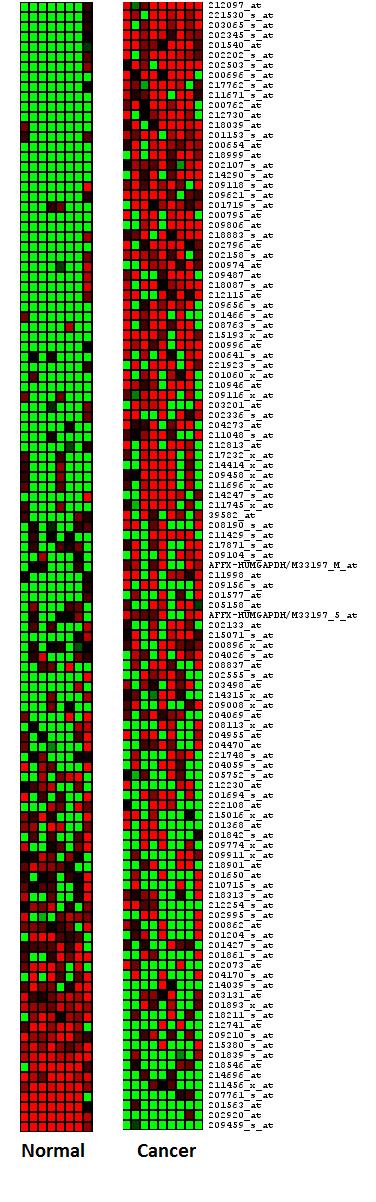

Supplement: Additional File 2 — The kidney cancer validation sets. Samples on the left are normal, right are cancer. The first image represents the Lenburg et al. [19] data set. The mislabelled samples in question are the rightmost 3 samples in the normal subgroup. [file 1471-2407-10-319-S2.JPEG]

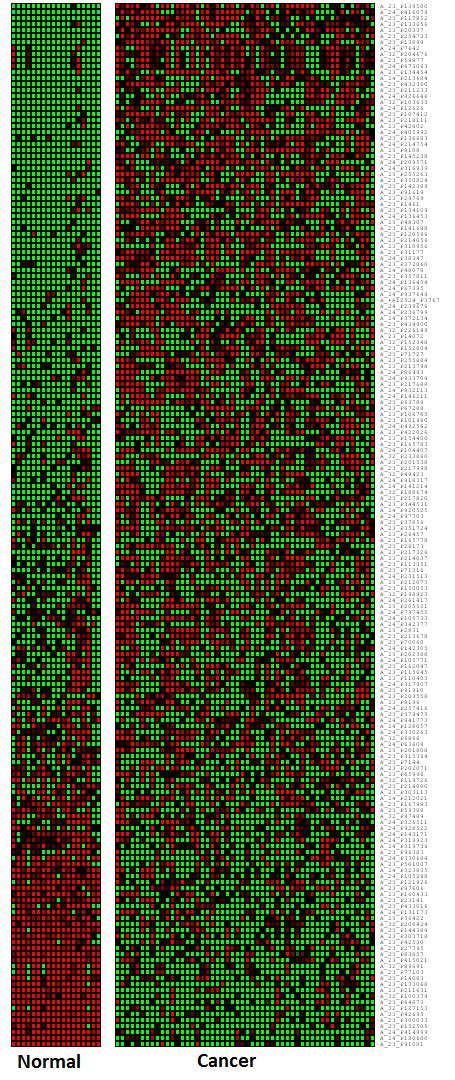

Supplement: Additional File 3 — ccRCC data from UNC labs. This image is of the ccRCC data [15,16] from UNC labs done on Agilent chips. [file 1471-2407-10-319-S3.JPEG]

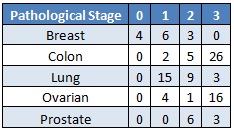

Supplement: Additional File 4 — Distribution of cancer stage in the training data. The following table shows the distribution of stages of the cancer samples in our training set. [file 1471-2407-10-319-S4.JPEG]
